# Toxicological data of some antibiotics and pesticides to fish, mosquitoes, cyanobacterial mats and to plants

**DOI:** 10.1016/j.dib.2016.01.051

**Published:** 2016-02-04

**Authors:** Yasser El-Nahhal, Nabila EL-dahdouh, Nisreen Hamdona, Adli Alshanti

**Affiliations:** Department of Envrionmental and Earth Science, Faculty of Science, The Islamic University-Gaza, Gaza Strip, Palestine

**Keywords:** Toxicity-data, Toxicity parameters, Mixture toxicity index, Relative toxicity, LC_50_

## Abstract

This article provides toxicological data of antibiotics to fish and mosquito (El-Nahhal and El-dahdaouh, 2015) (doi: 10.5132/eec.2015.01.03 [1]), to cyanobacteria (El-Nahhal and Alshanti, 2015)(dx.doi.org/10.4172/2161-0525.1000274 [2]) and pesticides to plants (El-Nahhal and Hamdona, 2015) (doi.10.1186/s40064-015-1148-7 [3]). The data provided herein described the experimental procedure and calculation of the appropriate toxicity parameters, lethal concentrations (LC_50_) required to kill 50% of tested animal, percentage growth inhibition, relative toxicity (RT) and Mixture toxicity index. Moreover, the data enable the readers to perform future experiments and open future discussion with other authors elsewhere and generate future research guidelines which benefit the young scientific community around the globe in the field of mixture toxicity.

**Specifications Table**

**Table:** 

Subject area	*Biology*
More specific subject area	*Eco-toxicology*
Type of data	*Table, photos, figure*
How data was acquired	*Laboratory* experiments encoded measuring mortality percentage; measuring optical density of cyanobacterial mat growth using colorimeter; measuring the physicochemical properties of the growth culture using eclectic conductivity meter, pH-meter and measuring plant heights using special ruler. (1) $\%\mathrm{Mortality}=100*(L_{c}-L_{t})/L_{c}$ *where L*_c_*and L*_t_*are average number of live animals in the control and treatment respectively .* % Growth inhibition = 100*(OD_c_−OD_t_)/ OD_c_*where* OD_c_*and* OD_t_*are the average optical density of the control and treatment respectively* Eq. (2).% Growth inhibition = 100*(Ph_c_−Ph_t_)/Ph_c_*where* Ph_c_*and* Ph_t_*are the average plant height of the control and treatment respectively* Eq. (3). Relative toxicity (RT) = LC_t50_/LC_s50_ Eq. (4),*where* LC_t50_*and* LC_s50_*are the toxicity parameters of tested and standard compounds respectively,* Eq. 4.
Data format	*Columns, treatments, groups, analyze.*
Experimental factors	*Breeding the experimental animals under the lab conditions for two weeks for acclimatization. Measurements of the lab conditions (e.g temp., Humidity, light/dark periods.), measuring individual and combined toxicity to fish mosquitoes, cyanobacteria and plants.*
Experimental features	*Measuring mortality % of fish and mosquitoes, the vitality of seeds, growth inhibition of cyanobacterial mats under lab condition to insure activity, vitality and validity for testing.*
Data source location	*Gaza-City, Palestine* (N, 31° 29′ 0.89″; E, 34° 24″ 3.08″).
Data accessibility	*Data is with this article.*

**Value of the data**

• The data provided here explain to the readers how they can easily calculate the toxicity parameters and use them to compare the effectiveness of tested compounds.
• The data also demonstrate visual rating of toxicity parameters, which can be a quantifying tool to measure the toxicity. In addition, it can also be a teaching tool that enables young researchers to effectively arrange their data.
• These data enable the reader to fully understand the environmental interactions of antibiotics and/or pesticides with the environmental biosphere.
• The data are useful enough to develop a biomarkers for detection of environmental contamination.
• The data also demonstrate different sensitivity of the tested organism in response to the same concentration of the tested compound. This enables the reader to explain various interpretations of the same toxic materials.

## 1 Data

**Fig. 1:**
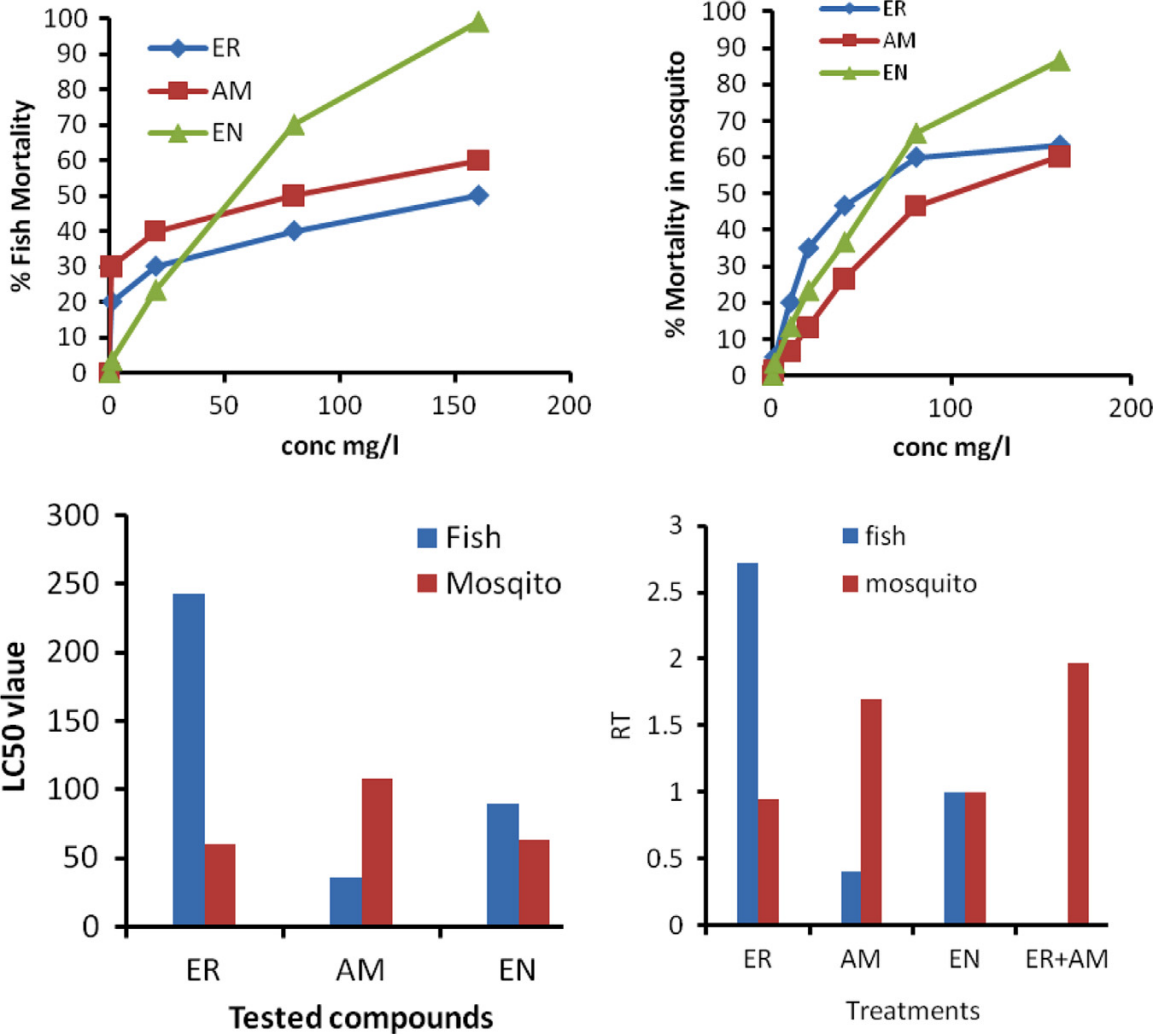
Responses of fish and mosquitoes to different concentration of antibiotics and standard toxic material (upper figures), estimated LC_50_ and RT values on fish and mosquito tests.

**Fig. 2:**
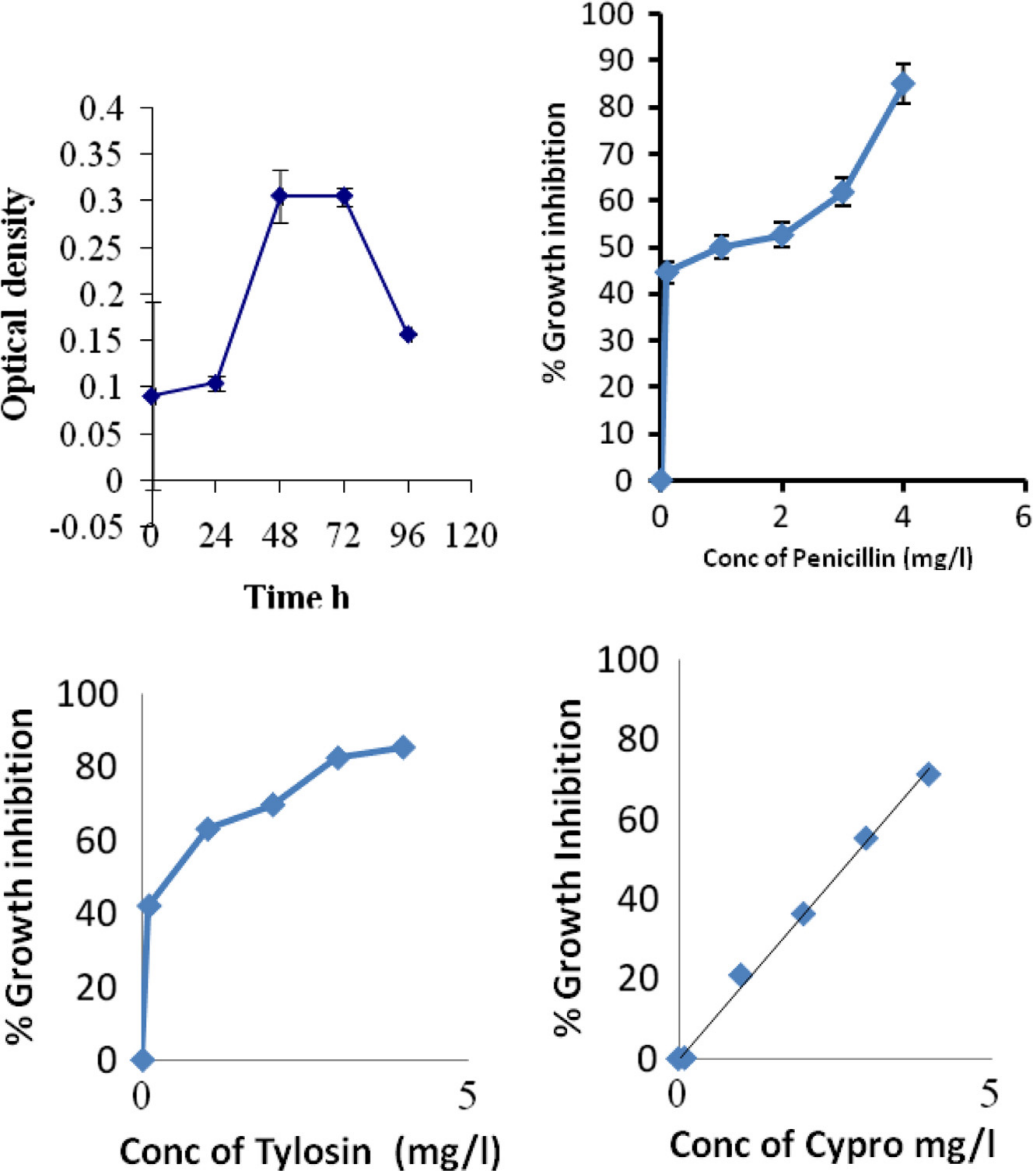
Four growth phases of cyanobacteria under laboratory conditions (upper case lift), upper right, Toxicity of Penicillin to cyanobacterial mats, lower cases are toxicity of Tylosin and Cyprophloxacin to cyanobacterial mats collected from Wadi Gaza.

**Fig. 3:**
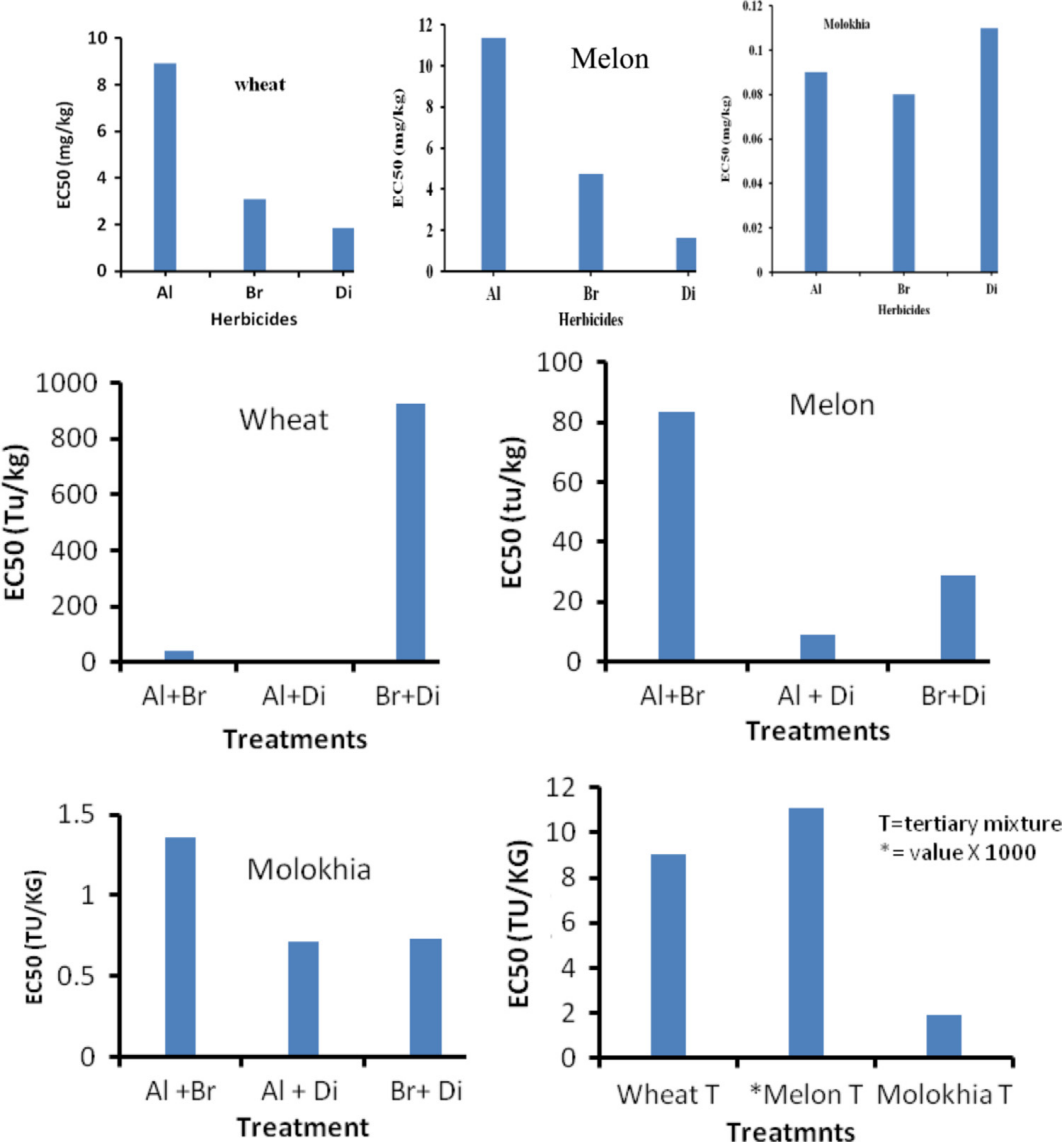
Toxicity parameters (EC_50_) of different tested compounds and their mixtures to the tested plants.

**Fig. 4:**
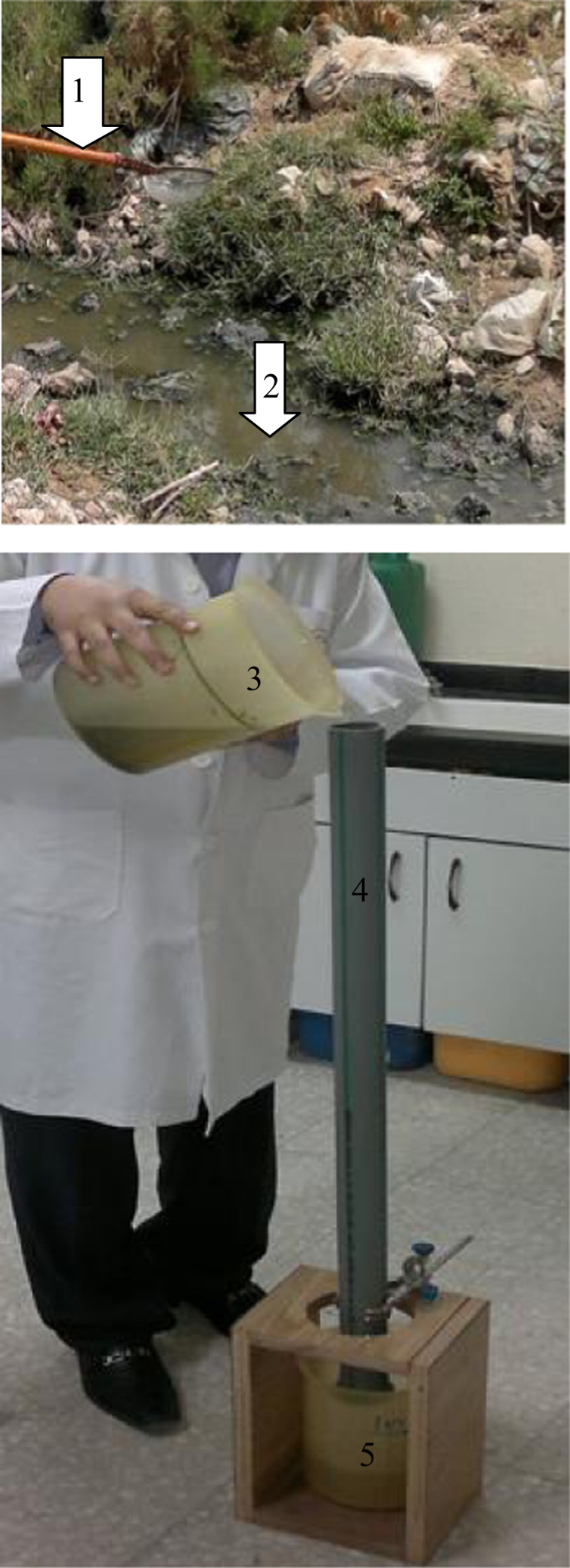
Cyanobacterial sampler collector (1), cyanobacterial mats (2), natural growth media of cyanobacterial mats (3), sand filter (4) and pure growth media (5).

The presented toxicological data (mortality percentage of fish and mosquitoes), lethal concentration required to kill 50% of tested organism (LC_50_), lethal time required to kill 50% of tested organisms, (LT_50_) relative toxicity (RT), effective concentration required to inhibit 50% of bacterial growth (EC_50_) (Fig. 1); growth phases, % growth inhibition (Fig. 2) herein give the reader an overview on the toxic responses of fish and mosquitoes to different concentrations of antibiotics (Erythromycin (ER) and amoxicillin (AM) and compared them with standard toxic material, Endosulfan (EN) ). The data in Fig. 2 also demonstrated the values of cyanobacterial mats growth phases under laboratory conditions and their responses to different concentrations of Penicillin, Tylosin and Ciprofloxacin as individual tests. Moreover, the data in Table 1 showed different responses (% growth inhibition) versus gradient concentrations of mixtures in toxic units, whereas the values of toxicity parameters (EC_50_, Regression equation (*R*^2^), and mixture toxicity index (MTI) are presented in Table 2. Furthermore, the data provided the responses of different plants (Melon, Molokhia and Wheat) to different herbicide concentrations of alachlor, bromacil and Diuron (Fig. 3) and demonstrated the EC_50_ values of single and mixture tests (Fig. 4). Furthermore, visual rating of toxicity provides an overview of plant responses to different herbicide mixture concentrations.

## 2 Experimental design, materials and methods

### 2.1 Toxicity test for fish

Following the procedure described in details in Ref. [1], fish larvae were collected from the farm to the laboratory and acclimatized under laboratory condition for 2 weeks at 25±2 °C before starting the toxicity tests. Fish larvae were transferred to 1 L glass beaker containing the required concentrations of the tested compound in the range of from 0–200 µg L^−1^. The experiments included positive and negative control samples. Mortality percentage (%) was recorded each 24 h up to 96 h. Toxicity was determined according to Eq. (1), Ref. [4].

### 2.2 Toxicity to mosquitoes

Mosquito larvae were collected from agricultural fresh water pond, transferred to the laboratory for acclimatization for 2 weeks at 25±2 °C before starting the toxicity tests.

The tests were performed in glass tubes (20 ml capacity) containing 10 ml of de-saline water having 10 larvae and the required concentration of the tested compounds in the range of 0–160 µg L^−1^. Number of dead larvae in each tube was observed and recorded every 24 h up to 48 h. Toxicity was determined according to Eq. (1) as shown above.

### 2.3 Toxicity to cyanobacterial mat

Cyanobacterial mat samples were collected from western part of Wadi Gaza using plastic bottles and algal net. Growth media (stagnant water) was collected from the same places of cyanobacterial mats growth, purified and cleaned up using sand filter developed in this study. The filtrate was autoclaved and used as growth media after cooling to room temperature. Cyanobacterial mats having optical density 0.16±0.03 at the starting time was allowed to grow under lab conditions, and the optical density was monitored each 4 h up to 120 h using CT-220 spectrophotometer at wavelength 680 nm [2]. The results of this experiment indicated the growth phases of cyanobacterial mats.

Toxicity tests were performed by preparing gradient concentrations of the tested compounds in the range of 0-16 mg/l and adding it to round bottom flasks containing 1 ml of cyanobacterial mat and growth media of total volume of 50 ml, the optical density of cyanobacterial mats suspension at the starting point was 0.16±0.03, in each flask [5]. The flasks were randomly arranged in the lab and optical densities of the flasks were recorded at 0, 24, 48, 72, and 96 h. The recorded data at 24 h were used to estimate the acute toxicity whereas, the recorded data at 72 h was used to estimate the chronic effect. According to Ref. [4], % growth inhibition (GI) which represents toxicity was calculated as follows:

(2) $$\%\;\left(\mathrm{GI}\right)=100*\left[\left({\mathrm{OD}}_{c}{-\text{OD}}_{t}\right){/\text{OD}}_{c}\right],$$

where OD_c_ and OD_t_ are the optical density of the control and the treated samples, respectively.

Regressing % growth inhibition [(OD_c_−OD_t_)/OD_c_] versus concentrations, enabled the estimation of EC_50_ values, where EC_50_ is the effective concentration that caused 50% growth inhibition compared with the control in any chosen toxicity endpoint. Detailed description of calculation is shown in Ref. [3].

### 2.4 Toxicity to plants

Following the procedure described previously Ref. [4] the toxicity tests were carried out with test plant in plastic pots under laboratory conditions using soil collected from agricultural area believed to be free of herbicides application at least in the past 5 years. In this test the required amount of the herbicides in the range of 0–1.7 mg kg^−1^ was taken from the stock solution and added to each plastic pot. Then the soil was mixed thoroughly in plastic bags to insure homogenized herbicide distribution. Then the soil transferred again to the plastic pots. Ten seeds of each crop were sown in each pot. Irrigated with 30 ml of fresh water and kept in the laboratory for 2 days. Then irrigated with 20 ml each day or whenever necessary. Plant heights were taken 2 weeks after germination and used as a parameter to measure the growth inhibition (%GI) according to Eq. (3) [4] and was taken as indicator of plant toxicity (phytotoxicity).

(3) $$\%\;\mathrm{Growth}\;\mathrm{inhibition}=100*\left(L_{c}{-L}_{t}\right){/L}_{c}$$

Where, *L*_c_ and *L*_t_ are the plant length (cm) in the control and the treatment at each measured concentration. Then %GI values were regressed with the tested concentration to calculate the LC_50_, the concentration required to inhibit 50% of plant growth. More details are shown in Ref. [3].

### 2.5 Binary mixture toxicity to plants

Binary or tertiary mixture solutions prepared according to Ref. [2] and diluted as follows (*M*/10,000, *M*/1000, *M*/100, *M*/10, *M*/5, and *M*), where*M* represents the original concentrations of mixture components. Then the diluted mixture solution were added to 1 kg air dried soil and mixed thoroughly to insure homogenized mixture concentration in soil. Then, the soil was transferred to the plastic pots for phytotoxicity evaluation as mentioned above. Percentage of growth inhibition of mixtures was determined as mentioned above.

### 2.6 Calculation of toxicity and statistical analysis

Relative toxicity (RT) was calculated according to Eq. (4).

(4) $${\text{RT}=\text{LC}}_{\mathrm{t50}}{/\text{LC}}_{\mathrm{s50}}$$

where LC_t50_ and LC_s50_ the lethal concentration of the tested and standard compounds, respectively. Value of RT equals or less than 1 indicates higher toxicity whereas value above 1 indicates lower toxicity than standard toxic substance.

Toxicity of mixtures was calculated based on % death and toxic units available in the solution. According to Sprague and Ramsay [6], toxic unit was calculated as follows:

Toxic unit=actual concentration in solution**/**lethal threshold concentration. Mixture toxicity index (MTI) was proposed by Konemann [7], to estimate synergism or antagonism of mixtures.

MTI=1−(Log*M*/Log*n*), where *M*=∑ *C*/EC_50_ at 50% effect in the mixture, and *n*=total number of compounds in the mixture.

Based on calculation, MTI value can be a negative (antagonism), zero (no effect) and positive value (synergism).

## 3 Responses of fish and mosquitoes to antibiotics

In Fig. 1 (upper cases), we presented data describing the responses of fish and mosquitoes to different concentrations of the tested compounds. Moreover, the magnitude of toxicity (LC_50_), (down case left), is different of each tested compounds. Furthermore, the left case showed the magnitude of RT, which is a measure required to judge the toxicity of the tested compounds.

## 4 Growth phases and responses of cyanobacterial mats to different antibiotics

In Fig. 2 (upper case left), we presented data describing the growth phases of cyanobacterial mats under lab conditions. The remaining parts of Fig. 2 demonstrated different percentage of growth inhibition due to exposure to different concentrations of penicillin, Tylosin and cyproflxacin. These data described different mode of action of the tested compound and different responses of cyanobacterial mats. Moreover, in Table 1 we presented data describing percentage growth inhibition of cyanobacterial mats to binary and tertiary mixtures. Furthermore, the toxicity parameters of binary and tertiary mixtures are presented in Table 2.

## 5 Toxicity to plants

In Table 3, we presented data describing the responses of melon, Molokhia and wheat to different herbicide concentration. Moreover, toxicity parameters (EC_50_) of different compounds and their mixtures are presented in Fig. 3.

**Fig. 5:**
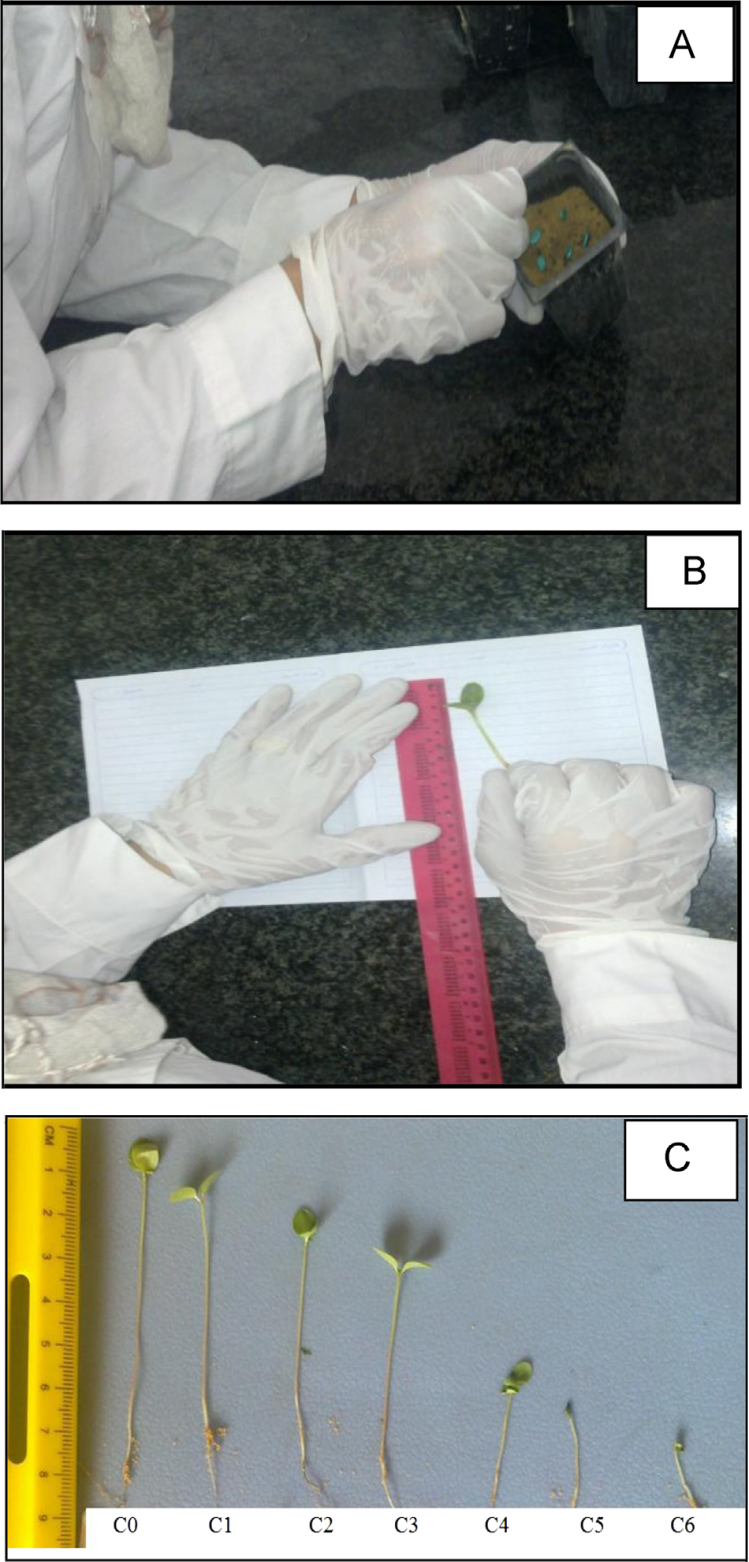
Visual phytotoxicity rating under laboratory conditions. A, B and C represent seeds emergence in pot experiment, plant height measurement and effects of alachlor concentrations (individual test) on Molokhia growth respectively.

Furthermore, data showing cyanobacterial mat sampler, floating mats, purification process of growth media using sand filter are presented in Fig. 4. In addition, data describing, planting, measuring plant heights and visual rating of phytotoxicity to Molokhia plants are shown in Fig. 5.

## Figures and Tables

**Table 1:** Percentage growth inhibition of cyanobacterial mats exposed to binary and tertiary mixtures of antibiotics.

Conc. (TU/l)	% Growth inhibition of cyanobacterial mats			
Penicillin 0.5+Tylosin 0.5	Tylosin 0.5+Ciprofloxacin 0.5	Pencillin0.5+Ciprofloxacin 0.5	Pencillin0.33+Tylocin0.33+Ciprof0.34
0	0.00	0.00	0	0
0.025	20.67	27.27	10.94	46.43
0.25	80.29	57.58	25	70
0.5	81.73	80.47	61.72	81.79
0.75	91.35	85.35	71.48	83.93
1	93.75	95.29	82.81	85

**Table 2:** Toxicity parameters of tested antibiotics.

Mixture content	Mixture type	EC_50_ (TU)	*R*^2^	Reg. Eq.	MTI
Penicillin 0.5+Tylosin 0.5	Binary	0.076	0.917	*y*=19.94+72.22	−3.70127
ciprofloxacin 0.5+Tylosin 0.5	Binary	0.103	0.966	*y*=41.612*X*+91.1	−2.29392
ciprofloxacin 0.5+penicillin 0.5	Binary	0.292	0.83	*y*=43.86*X*+73.46	−0.7918
Diuron 0.33+Diquat 0.33+terbutryn0.33	Tertiary	0.034	0.988	*y*=25.1*X*+86.71	−2.32488

**Table 3:** Phytotoxicity (% growth inhibition) of single herbicide to melon, Molokhia and wheat.

Conc. mg/kg soil	% Growth inhibition of Molokhia treated with		
alachlor	bromacil	diuron
0	0	0	0
0.005	2.86±0.48	9.77±0.53	0.23±0.90
0.01	18.57±0.39	10.36±0.60	5.25±0.92
0.02	21.43±0.65	29.72±0.62	22.66±0.78
0.075	32.65±0.27	50.11±0.38	31.14±0.76
0.1	55.36±0.56	60.72±0.34	40.14±0.39
0.15	64.29±0.32	63.72±0.36	43.66±0.35

0	0±	0±	0±
0.055	6±1.73	10±0.83	5±1.80
0.11	13±1.03	12±0.69	6±2.74
0.22	18±1.48	17±1.29	15±1.84
0.44	21±0.41	21±1.02	18±1.88
0.88	24±0.75	28±1.08	41±0.70
1.67	29±1.06	34±0.57	44±0.76

0	0	0	0
0.055	5±0.81	9±2.34	13±3.79
0.11	8±0.35	16±2.49	22±3.21
0.22	17±0.65	22±2.08	25±1.85
0.44	18±1.15	28±2.78	37±3.04
0.88	18±0.97	29±2.49	40±1.87
1.67	21±0.89	42±1.69	45±2.75
