# Supplementary material for: Toxicological data of some antibiotics and pesticides to fish, mosquitoes, cyanobacterial mats and to plants
Source: Data Brief. 2016 Feb 4;6:871–80. doi: 10.1016/j.dib.2016.01.051 (PMC4752731; doi:10.1016/j.dib.2016.01.051)
Supplement: Supplementary file 1 — Supplementary material [file mmc1.doc]

Conflict of Interest Form

**We are:**

**El-Nahhal Yasser, EL-dahdouh Nabila, Hamdona Nisreen, Alshanti Adli**

**Authors of** Data article Manuscript No.: DIB-D-15-00374

Entitled: Toxicological Data of Some Antibiotics and Pesticides to Fish, Mosquitoes, Cyanobacterial Mats and to Plants

**We declare no conflict of interest with any body or organization and agree to have our manuscript published in DIB journal.**

**Please consider this as an official document of declaration.**
